# Supplementary material for: Are carbon emissions trading and green financial instruments synergistic? -Comprehensive quantitative research based on content analysis
Source: PLoS One. 2024 Mar 7;19(3):e0298601. doi: 10.1371/journal.pone.0298601 (PMC10919601; doi:10.1371/journal.pone.0298601)
Supplement: S1 Data — (ZIP) [file pone.0298601.s001.zip › Data/地级市Z_F工作报告.zip!/╔╜╢1⁄2/╝├─╧╩╨/└┤╫╘╣1⁄2╓┌║┼í╛┬φ┐╦╩2╛▌═°í┐_2016.docx]

各位代表：

　　现在，我代表市人民政府向大会报告工作，请予审议，并请各位政协委员提出意见。

一、2015年及“十二五”工作回顾

　　2015年是“十二五”规划收官之年，也是我们主动适应新常态、引领新发展的重要一年。一年来，市政府认真贯彻落实党中央国务院、省委省政府和市委各项决策部署，围绕“打造四个中心，建设现代泉城”中心任务，全面执行市十五届人大四次会议决议，统筹推进稳增长、促改革、调结构、惠民生、防风险各项工作，全市经济社会发展取得新的成就。预计实现生产总值6200亿元，增长8%；一般公共预算收入614.3亿元，增长13.1%；固定资产投资3500亿元，增长14.2%；主要指标增幅均达到或超过全省平均水平。经过全市戮力攻坚，我们成功创建了国家卫生城市和国家森林城市，成为海绵城市首批国家试点城市和国家生态文明先行示范区。全市经济运行总体平稳、稳中有进，社会发展文明向上、持续向好。一年来，主要做了以下工作。

　　一是激活力、强实体，实现经济新增长。面对前所未有的经济下行压力，我们把稳定经济增长作为政府工作首要任务，精准施策，聚焦发力，及时推出“稳增长50条”、“财政28条”、县域经济差别化扶持政策等一系列措施办法，着力解决审批、建设、融资、征地拆迁、创新创业等环节制约性难题。集中力量推进项目建设、招商引资和棚改旧改工作，实施项目建设提升计划，建立领导包挂项目推进机制，开展重点项目建设督查评议活动，启动了齐鲁制药产业园、传化泉胜公路港、百步亭等一批高端高质产业项目，推进220个市级重点项目投资1110亿元。多措并举破解融资难题，加大融资担保、增量贷款财政补贴力度，争取地方政府债券206.9亿元、国家开发银行棚改贷款预授信300亿元，在全省率先推出17个PPP项目,引导社会资本投入基础设施、重点产业、社会事业等领域。出台新一轮国有土地上棚户区改造意见，扩大货币化安置，加快房地产去库存，商品房销售面积增长37.7%。全力扩大消费需求，建设投运了方特东方神画、济南野生动物世界等一批拉动旅游、吸引消费的服务业载体项目，加快发展健康、养老、信息、文化、教育等消费服务，完成社会消费品零售总额3407.5亿元，增长10.4%。实施扶持外贸稳定增长一揽子政策措施，为企业解决通关、退税等70多项难题，出口增幅高于全国、全省平均水平，境外实际投资5.6亿美元、增长160%，对外承包工程营业额23.4亿美元、增长34.7%。

　　二是抓调整、促转型，催生产业新引擎。坚定不移推进产业深度调整和创新驱动，成立重大产业发展协调推进领导小组，出台实施电子商务、旅游业、服务业、生物医药、电子信息等支持措施，着力培育新产业新动力，经济质量和效益有了新提升。服务业比重达到56.5%，提高0.7个百分点；服务业税收占全部税收的60.8%，提高1.26个百分点；高新技术产业产值比重达到42.58%，居全省首位；信息软件、生物医药、高端装备等产业投资均增长28%以上。一批新兴产业项目投产达效，成为新的增长点。继齐鲁制药之后，力诺成为我市第二家过百亿民营企业。云计算产业集群获得省重大专项立项，引进培育无人机、工业机器人、智能电网、创意设计、数字传媒等领域一批新兴企业。建筑产业化应用面积达到790万平方米。创新谷、药谷、新材料产业园等载体建设加快推进。全市各类科技孵化器、生产力促进中心达到50余家，孵化面积100万平方米、在孵企业1400余家，扶持打造21家“泉城众创空间”。实施一批重大科技专项，浪潮“高端容错计算机系统关键技术与应用”、宏济堂“人工麝香研制及其产业化”荣获国家科技进步一等奖，二机床大型先进智能冲压设备实验室成为国家重点实验室，圣泉集团被评为国家技术创新示范企业，九阳豆浆机获得中国发明专利金奖，成立山东工业技术研究院。新业态新模式迅速成长，电子商务交易额达到2180亿元，增长35%；互联网金融、股权投资、融资租赁等现代金融业态实现新的发展。新增上市公司3家、新三板挂牌企业47家，山东金融资产交易中心交易量突破100亿元。

　　三是补短板、促统筹，推动城乡新发展。推进旧城更新和新区开发，加快中央商务区、华山、雪山、北湖、南北康、西客站、东客站等重点片区拆迁建设，完成4.6万户棚户区改造任务。清雅居公租房项目荣获国家鲁班奖。编制完成历史文化名城保护以及城市色彩、地下空间、绿化等20多个专项规划。重点实施一批水电气暖、信息网络等公共服务设施建设，新增集中供热面积1052万平方米，改造水气热危旧管网271公里，全市商务楼宇光纤接入和公共热点区域无线网络接入实现全覆盖。加快交通路网建设，轨道交通R1线全面开工，二环南路快速路西段建成通车，完成34条市区道路建设改造任务，济南长途汽车西站正式启用，开通市区至长清、济阳等9条公交线和济莱、济齐城际快客。纬十二路、泺文路等人防工程顺利推进。加强生态环境建设，实施大气污染防治“十大行动”，突出燃煤、尾气、扬尘、工业生产综合治理，加快工业余热利用，扎实开展海绵城市创建工作，实施玉符河综合治理、地表水转换地下水、五库连通、渗漏带修复等工程，山体生态修复暨山体公园建设项目获得中国人居环境范例奖。趵突泉群保持12年持续喷涌。我市被评为国家防震减灾示范城市。

　　农业现代化稳步推进，粮食实现“十三连丰”。现代农业园区和粮食生产功能区加快建设，规模以上农业龙头企业、农民合作社、家庭农场分别达到536家、5608家和1276家。电商下乡、乡村生态游、观光农业成为拉动乡村经济新业态。完成一批水库除险加固工程，建成“五小水利”569处。万德、刁镇、孔村等17个国家重点镇、省市级示范镇承载集聚力增强。一批开发区扩围培育新产业，成为县域发展新载体。深入推进美丽乡村示范村创建和乡村连片治理，开展“千村五化提升”行动，建设农村公路506.5公里。建成省级以上生态镇59个、省级生态村53个、市级文明生态村3300个。实施农村扶贫开发项目427个，惠及465个村6万贫困人口。解决了22.62万农村居民和7.55万学校师生饮水安全问题。

　　四是促改革、扩开放，培育竞争新优势。发布施行行政权力清单、责任清单，深化行政审批“两集中两到位”改革，全面实施“三证合一、一照一码”登记制度，完成政府部门行政审批中介服务脱钩任务。创新招商引资体制机制，组建投资促进局。启动高新区、新材料产业园体制机制创新试点。市县不动产统一登记职责和机构整合完成。探索实施公务员聘任制和事业单位分业分类招聘办法。全部公开市县两级财政预算、部门预算及“三公”经费预算，市本级“三公”经费预算下降8.99%，收回结余沉淀资金16.13亿元全部用于社会民生。推进产业引导资金股权改革，设立济南财金投资发展基金，首期募集100亿元。将2015年国有资本经营收益10%调入一般公共预算。理顺城市供水体制，实现制水供水企业合并。实行居民用水、用气阶梯价格制度。农村土地确权登记颁证基本完成，农村综合产权流转交易体系建设全面启动。顺利实施机关事业单位养老保险、医疗保险改革。我们还按照市委十届八次全会部署要求，坚持问题导向，解放思想，长短结合，启动了对影响济南发展若干重大问题的研究，一些改革措施已经付诸实施。

　　开放型经济稳步发展。开展一系列境内外招商洽谈活动，引进宜家、麦德龙等国际知名企业落户我市。利用外资15.8亿美元，增长10%。跨境电商平台上线测试，进口商品保税展示交易中心正式运营，完成济南机场“三个一”通关试点。成功举办第四届韩博会。中日韩产业合作济南先行示范区建设启动，综保区、明水开发区与韩国光阳湾圈经济自由区签订合作协议，中韩尖端产业融合园进入策划实施阶段。济莱协作区交通、旅游、医疗协作取得积极进展。

　　五是惠民生、促和谐，增创市民新福祉。全面完成为民办18件实事（附件1）。一般公共预算民生支出比重达到75.1%，提高0.7个百分点。城乡居民人均可支配收入分别达到39876元和14317元，增长8%和9.2%。居民消费价格涨幅为1.9%。大力扶持创新创业，新增城镇就业20.73万人，农业富余劳动力转移就业5.6万人，阳光大姐等平台就业促进作用增强。各项社保基金征缴总收入创历史新高，连续11年提高企业退休人员养老金，月人均达到2633元，居民养老保险基础养老金实现“五连涨”，城镇低保和农村低保标准分别提高到每人每月550元和300元，发放残疾人生活救助金1.2亿元。做好国有困难企业帮扶解困工作，累计发放资金14.94亿元、惠及职工3.67万人。加快校舍建设改造，开工建设中小学46所，新建改建扩建幼儿园90处，实现了义务教育阶段“零择校”。完成县级公立医疗机构改革，获评全国基层中医药工作先进单位。“单独两孩”政策平稳实施。文化惠民活动深入开展。中国非物质文化遗产博览会永久落户济南，各类博物馆、纪念馆突破180座。圆满承办第22届国际历史科学大会。开展纪念中国人民抗日战争暨世界反法西斯战争胜利70周年系列活动。新开工建设保障性安居工程51168套，竣工8935套。推进社会治理创新,开展“三区三圈”治理，形成立体化社会治安防控体系。开展国家食品安全城市创建试点工作，食药监管、安全生产形势稳定向好。

　　一年来，我们自觉接受人大依法监督、政协民主监督和社会监督，认真办理人大代表议案、建议和政协建议案、提案,支持民主党派、工商联和无党派人士参政议政。开展法治城市创建，深入实施“六五”普法和法制宣传教育，加快公共法律服务体系建设。加强依法行政和法治政府建设，建立政府法律顾问队伍，推行行政单位负责人应诉制度。践行“三严三实”，开展“直面问题践行承诺”活动，狠抓群众意见建议和中央、省委巡视组反馈意见整改落实，加大对不作为乱作为问责力度，电视问政、“啄木鸟在行动”等活动受到社会好评。市政务服务中心建成运营，实现多平台集中便民服务。严格落实党风廉政建设责任制，推进政府系统惩治和预防腐败体系建设，反腐倡廉取得新成效。侨台、民宗、慈善、老龄、妇女儿童、红十字、档案、史志、气象、文物保护、双拥共建以及援藏援疆等工作都取得新的进步。

　　各位代表！

　　2015年各项工作任务全面完成，标志着我市“十二五”规划圆满告竣。过去的五年，面对复杂严峻的经济形势和紧迫繁重的改革发展任务，我们发扬顽强拼搏的精神，集中力量解决了一批前所未有的难事，办好了一批群众欢迎的实事，办成了一批具有深远意义的大事，省会改革开放和现代化建设取得新的重大成就。

　　——过去的五年，是综合实力显著增强、产业结构深度调整、发展质量持续提升的五年。2015年，全市生产总值是2010年1.6倍，年均增长9.3%，人均1.4万美元；一般公共预算收入是 2010年2.3倍；固定资产投资五年累计13322亿元，是“十一五”时期1.8倍；金融机构本外币存贷款余额分别突破1.5万亿元和1.1万亿元，是2010年2倍和1.6倍；服务业比重、高新技术产业产值比重分别提高3.9个和5个百分点。提前完成节能减排指标任务。一批承载高端产业、集聚人才技术、极具倍增潜力的增长板块正在崛起，初步构筑起服务经济优势主导、新产业新业态加速发展的省会现代产业体系。

　　——过去的五年，是城市空间不断拓展、服务功能日臻完善、城乡面貌明显改观的五年。全市建成区面积500平方公里，户籍城镇化率62%。建成一批城乡供水、供电、燃气、供热、排水、排污设施。城市快速交通路网初步形成。小城镇、农村新型社区建设扎实推进。森林覆盖率、建成区绿化覆盖率分别提高到35.2%和40.2%。泉城特色与水系生态水平不断提升。南部山区生态保护与北部跨河发展正蓄势进入新阶段。城市更新和功能拓展继续深化，以一城三区为核心、以经十路东西延展为轴线、片区园区联动、城乡统筹发展的新格局已经形成。

　　——过去的五年，是改革开放持续深化、体制环境不断优化、发展活力继续增强的五年。全面深化改革迈出坚实步伐，有序推进了行政审批、财税金融、国资国企、农业农村和社会治理等重点领域改革，审批收费事项大幅精简，市场准入门槛降低，营商环境明显改善，创新创业蔚然成风。全市各类市场主体达到47.8万家。综保区、明水开发区升级国家队，济南海关升格为总署直属海关，增强了开放发展承载力。新增6个友好城市和18个友好合作城市，国际友城布局全面优化，对外交流合作成效明显。省会城市群经济圈一体化进程加快。

　　——过去的五年，是生活质量稳步提升、社会保障不断加强、社会事业全面进步的五年。五年来,全市从一般公共预算收入中安排1914亿元用于改善民生和发展社会事业,是“十一五”时期2.5倍。城乡居民收入比2010年分别增长65.3%和80.6%。新增城镇就业和农业富余劳动力转移就业122.2万人。建设保障性安居工程14.3万套。城乡低保标准分别提高37.5%和100%，养老、医疗等社会保障水平大幅提高。初步构建起覆盖城乡的公共服务体系，各项社会事业有了很大发展。成功举办第十届中国艺术节、第三届非物质文化遗产博览会等重大活动，泉城历史文化品牌日益彰显，群众性文化生活丰富多彩。社会治理不断加强，安全生产形势平稳，治安防控能力增强，社会大局和谐稳定。

　　各位代表!五年来的巨大发展和进步，是我们全面贯彻落实党和国家路线方针政策的结果，是省委、省政府和市委坚强领导的结果，是人大、政协监督支持的结果，是全市共同努力的结果。在此，我代表市人民政府，向全市各族人民、各界人士、驻济部队、武警官兵和中央、省驻济单位，向港澳台同胞、海外侨胞和国际友人，表示诚挚感谢和崇高敬意!

　　我们也清醒地认识到，我市发展中还存在不少困难和问题，政府工作还有许多不足。经济增长内生动力不够强，投资增长乏力，创新能力不足；传统产业转型、新兴产业发展不够快，企业生产经营困难增多；县域经济薄弱、区域发展不平衡的问题仍旧突出；实现基本公共服务均等化面临不少瓶颈制约，群众对民生工作还有不少不满意的地方；雾霾频发、交通拥堵尚未得到根本缓解；重点领域改革难度较大，社会对改善营商环境仍有较高期待；有些政府工作人员担当力不强，不作为、乱作为和消极腐败现象依然存在。我们一定正视问题、标本兼治，不以事艰而退避，不以任重而不为，采取有力措施，认真改进和解决。

二、“十三五”时期的主要目标和任务

　　根据《中共济南市委关于制定济南市国民经济和社会发展第十三个五年规划的建议》，我们编制了《济南市国民经济和社会发展第十三个五年规划纲要（草案）》，提交大会审议。

　　“十三五”时期是全面建成小康社会的决胜阶段。我国已进入新常态攻坚发展的新时期，新型工业化、信息化、城镇化、农业现代化、绿色化“五化”协同发展，经济长期向好的基本面没有改变。从我市看，全市发展已经迈上了新的起点，我们既面临改革创新、转型升级、开放发展所蕴蓄的重大契机，也面临稳定增长、补齐短板、均衡发展、改善生态的紧迫任务。总体上看，“十三五”时期我市仍处于可以大有作为的重要战略机遇期。

　　我们要全面贯彻党的十八大和十八届三中、四中、五中全会精神，深入贯彻习近平总书记系列重要讲话精神，按照中央“四个全面”战略布局和省委、省政府决策部署，紧紧围绕“打造四个中心，建设现代泉城”中心任务，坚持发展是第一要务，以创新、协调、绿色、开放、共享发展新理念为引领，以提高发展质量和效益为中心，统筹推进经济建设、政治建设、文化建设、社会建设和生态文明建设，确保在全面建成小康社会进程中走在全省前列，奋力开创省会现代化建设新局面。

　　——坚持创新聚焦，全力打造“四个中心”。今后五年经济发展主要预期目标是：生产总值年均增长8%，一般公共预算收入年均增长9%，固定资产投资年均增长13%，服务业比重提高到60%。我们要打造全国重要的区域性经济中心，加快产业更新和动力转换，积极对接“中国制造2025”战略，实施“互联网+”重大产业工程，加快培育高端高质高效现代产业体系，经济影响力、区域带动力和环境吸引力大幅提升。我们要打造全国重要的区域性金融中心，举全市之力建设中央商务区，建设区域性金融管理中心、金融机构中心、资金结算中心、金融交易中心、金融后台服务中心以及金融机构集聚发展高地，金融服务实体经济能力大幅提升。我们要打造全国重要的区域性物流中心，创建综合运输服务示范城市，建设一批网络化、规模化、信息化的物流园区、物流基地和骨干企业，构建开放高效生态智慧的现代物流体系，2020年实现社会物流总额3.5万亿元。我们要打造全国重要的区域性科技创新中心，推动大众创业万众创新，新技术、新产业、新业态蓬勃发展，高新技术产业产值比重达到46%以上，初步建成国内重要的科技成果策源地和京沪之间科技创新新高地，率先建成创新型城市。

　　——坚持协调融合，加快建设现代泉城。牢固树立精明增长理念，突出产城融合和城乡联动，推动城市建设转型升级。着力优化发展布局，推进城市更新，拓展发展空间，建设形成中心、次中心、卫星城以及重点镇、一般镇梯次分布、互相衔接、功能完善的城乡一体化发展布局。规划建设携黄河两岸融合发展的城市新区。加强南部山区生态保护，确保南部山区森林覆盖率达到并稳定在70%以上。协同推进新型城镇化和新农村建设、现代农业和县域经济发展，户籍人口城镇化率提高到67%，县域经济比重提高到45%。统筹推进交通、能源、通信、水利、安全保障、地下空间等基础设施建设，城市综合承载力和可持续发展能力显著增强。

　　——坚持绿色低碳，展现生态泉城新形象。围绕建设国家生态文明先行示范区，完善生态文明法规体系，健全生态补偿机制，强化环境监管执法，着力解决环境污染突出问题，推动低碳循环发展，加快建设资源节约型、环境友好型社会。完善优化开发、重点开发、限制开发和禁止开发的主体功能区规划。坚持节约优先，发展循环经济，促进资源集约高效利用，到2020年全市能源消费总量控制在4800万吨标准煤以内，划定永久基本农田，守住耕地红线。坚决打好治霾攻坚战，努力破解交通拥堵难题，全面提升省会宜居宜业宜游水平。坚持科学保泉，弘扬泉水品牌，统筹推进水生态文明市和海绵城市建设，加强历史文化名城保护，打造百里黄河生态画廊，完成126座山体绿化，建设天蓝、地绿、水清、泉涌的生态泉城。

　　——坚持改革开放，不断增创发展动力和空间。全面深化以经济体制改革为重点的各项改革，构建多元开放格局，激活发展内生动力。深化行政管理体制改革，进一步转变政府职能，建设法治政府、创新政府、廉洁政府和服务型政府。深化国资国企改革。大力发展民营经济。完善土地、资本、人才等要素建设，加快形成开放有序的现代市场体系。借鉴上海自贸区创新制度，打造法治化、国际化、便利化营商环境。优化市场布局和贸易结构，加快发展服务贸易和服务外包，推动外贸转型升级。将招商引资作为经济工作生命线，确保现代服务业、战略性新兴产业引资比重每年提高1-2个百分点。健全“走出去”服务保障体系，鼓励我市企业积极开展跨国并购和投资，推动优势产能对接融入国家“一带一路”建设。在科技创新、基础设施、产业协作、污染治理等领域与周边城市加强合作，加快省会城市群经济圈建设步伐。

　　——坚持共建共享，全面改善人民群众生活。完善制度安排、强化政策兜底、统筹社会资源，扎实做好各项民生工作，实现全市人民共同迈入全面小康社会。实施精准扶贫精准脱贫工程，确保2018年全市现行标准下农村贫困人口全部脱贫。推动义务教育均衡发展，全面普及高中阶段教育，构建现代职业教育体系，不断提高教育质量。坚持就业优先战略，完善以创业带动就业的扶持政策，统筹推进各类群体创业就业。建设健康济南。完善养老服务机制，实施全民参保计划，形成覆盖全民、整合城乡、协调均衡的社会保障体系。到2020年城乡居民人均可支配收入分别提高到58600元和21500元以上。全面提升公共文化服务，着力打造文化泉城。促进人口均衡发展，加强和创新社会治理，确保社会和谐稳定。

　　总之，经过未来五年努力，全市经济更加发展，城乡更加繁荣，环境更加美好，人民更加幸福，制度更加完善，社会更加文明，打造全国重要的区域性经济中心、金融中心、物流中心、科技创新中心和建设享誉中外的现代泉城取得重大进展，提前完成泉城市民共同期盼的全面小康社会宏伟目标。

三、2016年的工作

　　今年是“十三五”规划开局之年，也是本届政府收官之年，更是“打造四个中心，建设现代泉城”破题起势之年。做好今年工作意义重大。按照中共济南市委十届八次、九次全会部署和“十三五”规划纲要要求，今年经济社会发展主要预期目标是：生产总值增长8%左右，一般公共预算收入增长9%左右，固定资产投资增长13%左右，社会消费品零售总额增长11%左右。农业、服务业、规模以上工业增加值分别增长4%、8.5%和8%左右，高新技术产业产值比重提高1个百分点。进出口增长保持稳定，实际到账外资增长6%左右。城乡居民人均可支配收入分别增长8%和8.5%左右，居民消费价格涨幅控制在3%左右，城镇登记失业率控制在4%以内。全面完成省政府下达的年度节能减排约束性指标。

　　当前，国内外经济形势依旧错综复杂，经济下行压力持续增大，稳增长、促改革、调结构、惠民生、防风险任务艰巨。在今年政府工作中，必须牢固树立和贯彻落实创新、协调、绿色、开放、共享发展理念，坚持稳中求进工作总基调，着力推进供给侧结构性改革，全面贯彻去产能、去库存、去杠杆、降成本、补短板的要求，把握稳增长和调结构的平衡，做好产业更新和动力转换两篇大文章，集中优势力量，聚焦关键问题，千方百计增加有效投资，不断创造新需求新动力，全面推进“打造四个中心，建设现代泉城”各项工作，确保在关键环节取得突破性进展，确保实现经济稳定增长，努力以结结实实的业绩开创“十三五”良好开局。

　　 (一)全力推进“四个中心”建设，加快动力转换产业更新。全面实施“三年行动纲要”，聚力攻坚，落地抓实，确保“四个中心”建设一年有势头、两年有看头、三年有突破。

　　全力推进区域性经济中心建设。以智能制造、“互联网+”为引领，突出优势产业、龙头企业和重点项目，融合发展先进制造业和现代服务业，力争用三年时间新增规模以上工业企业300家、服务业企业200家以上，打造“济南设计”、“济南智造”、“济南服务”品牌。坚持领导包挂重点产业，实施一链一策、优势带动，研究推出分业扶持政策措施，对重点产业和龙头企业采取特惠制，招商推进一批重大产业项目，厚培壮大交通装备、电子信息、机械装备等千亿级产业集群，培育发展新一代信息技术、生物医药及高性能医疗器械、节能与新能源汽车、新材料、新能源、建筑产业化等新兴优势产业。把发展智能制造和“互联网+”作为济南工业弯道超越的引擎和重大机遇，推出《中国制造2025》济南实施方案，出台智能制造产业五年发展规划，建设东部、西部两大智能制造研发服务平台，实施“机器换人”示范工程，推进高档数控机床和机器人产业化与应用。扶持发展互联网经济，开展产业创新、公共服务等17项专项行动，实施互联网与工业融合创新示范工程，开展大数据增值性、公益性开发和创新应用。实施一批技改创新项目，推进钢铁、化工、建材、纺织、食品等产业绿色改造，促动传统产业转移升级。以研发设计、检验检测、成果转化服务为重点，打造一批生产性服务业和高技术服务业集聚区，加快生产制造向“生产+服务”转型。深化实施“五个一批”梯度培育工程，加强12个国家火炬特色产业基地建设，促进中小微企业和特色产业做大做强。实施质量强市、名牌战略和标准化战略，加强知识产权创造、运用和保护。

　　全力推进区域性金融中心建设。举全市之力建设中央商务区，一同推进规划、拆迁、建设、招商各项工作，围绕新总部经济、“金融+”和现代商务服务业，抓好核心区、起步区引爆项目、超高层项目落地开工，策划十大金融招商项目，打造高端业态和总部经济集聚区。做大做强本地法人金融机构，支持齐鲁银行、济南农商行综合混业发展，鼓励泰山、安顾等保险机构发展壮大，扶持山东省城市商业银行合作联盟加快发展。加快山东金融资产交易中心、全景网山东路演中心等平台发展，支持更多企业上市挂牌融资。培育互联网金融、股权众筹、私募基金、融资租赁、金融中介服务等新兴金融业态，吸引上市公司或大型企业在济设立研发总部、租赁公司、财务公司，促进山东新金融中心、汉峪金谷错位联动发展。筹建金融投资控股集团。引导金融机构加大对市域实体经济信贷投放，加快发展农村金融等普惠金融,鼓励保险资金参与我市基础设施建设、棚户区改造。规范各类融资行为，有效防控金融风险。

　　全力推进区域性物流中心建设。规划建设桑梓店物流中心、董家公铁货运中心、空港国际物流中心三大枢纽及崔寨、大桥路两大综合园区，加快大舜医药物流、新材料仓储交易中心、丰树仓储物流园等21个重点物流项目建设，改造提升零点物流港、盖世物流园，支持佳怡等第三方物流和韩都衣舍等新兴物流企业发展壮大。加快省会城市群经济圈一体化信息平台及保税区跨境电商信息平台建设，推进冷链、农产品交易等一批物流信息平台建设，大力发展城市配送、农村物流、快递物流、医药物流等特色物流。启动“济新欧”国际货运班列前期工作，争创国家级多式联运示范工程。

　　全力推进区域性科创中心建设。完善高新区体制机制，拓展发展空间，高标准建设智能装备城、生命科学城、齐鲁智慧谷、齐鲁创新谷“两城两谷”，积极争创国家自主创新示范区，打造辐射周边的创新引领新高地。进一步完善全市科技创新管理体制机制。实施企业能力提升工程，加大对领军企业、“小巨人”、“金种子”企业扶持力度，落实高新技术企业税收优惠、研发费用加计扣除等激励政策，开展规模以上工业企业研发投入“清零”行动。推进国家信息通信国际创新园、国家重大新药创制平台、浪潮高性能计算中心、山东量子技术研究院等重大源头创新平台建设，力争在重大创新领域创建国家实验室。推动大型仪器共享平台建设，开工建设山东工业技术研究院基地，筹建山东复旦研究院济南成果转化中心，加快济南—德国科技创业中心建设，组建一批产业技术创新战略联盟。设立股权投资母基金和天使投资、制造业上台阶、新三板提升等子基金，建设一批科技金融特色银行。打造泉城科创交易大平台。新增众创空间28家，允许众创空间“席位注册”、“一址多照”。强化“齐鲁人才特区”建设，更多引进“5150”等高层次人才，完善海外人才来济创新创业服务保障机制，实施推动科技人员服务企业与推动科技人员自主创业“双推”工程。大力营造尊重企业家和创业者的良好社会氛围。

　　(二) 狠抓三项重点工作，着力扩增量稳增长。项目建设、招商引资和棚改旧改是“四个中心”建设的关键环节。全市各级各部门必须按照三项重点工作责任书，埋头苦干，狠抓落实，着力稳增长蓄后劲打基础。

　　全力以赴抓好项目建设。继续实施项目建设提升计划，突出重大基础设施、新兴产业、科技创新、金融物流、棚改旧改、生态环境、社会民生等重点领域，推进落实总投资3310亿元150个市级重点项目，年计划投资768亿元，其中新开工项目76个、年计划投资309亿元。继续抓好华山、北湖、雪山、南北康等22个重点片区开发建设。加快落实首批PPP项目，围绕交通、环保、医疗等领域继续推出一批新项目，完善扶持机制和优惠政策，力争三年内政府和社会资本合作项目总投资达到1000亿元。实行重点项目“预备库—储备库—建设库”三库联建，制定实施政府投资项目三年滚动投资计划，完善重大项目动态调整机制，确保三年内重点项目总投资达到1万亿元。实施重点项目推进十大机制，倒排工期、挂图作战，推动项目建设提速提效。积极争取国家和省在项目规划布局、要素保障、专项建设债券发行等方面更大倾斜，深入开展银企对接活动，做好授信协议的跟进落实。

　　多措并举抓好招商引资。开展“投资促进年”活动，创新引资引技引智机制，统筹推进全市招商引资工作。一是突出重点产业招商，围绕我市主导产业和优势新兴产业，以重汽、浪潮、二机床等重点企业为龙头，策划引进配套企业和项目，促进产业延伸集聚、做大做强。二是突出重点板块招商，各发展主体按照全市产业统一布局，以园区、片区、楼宇为载体，抓好中央商务区、汉峪金谷、创新谷、中新智慧城等重点片区招商，引进一批高端产业，培植一批亿元楼宇，打造一批增长高地。三是突出重点企业招商，瞄准世界500强、国内500强、民营500强，引进大项目好项目特别是引爆型项目落地发展,加快中弘旅游、万达鲁秀、中广核产业协作等一批项目进度。四是突出重点区域招商，把握国内外产业和要素流动新趋势，策划德国工业4.0和美国工业互联网领域专题招商，开展赴美国、德国、韩国、新加坡、香港、台湾以及北京、上海、珠三角等境内外重点区域招商推介活动，组织中德中小企业高峰论坛和中美财富论坛。探索“互联网+招商”、市场化招商新模式，建立招商项目库、产业专家库、信息数据“云后台”，强化跟踪服务，增强招商引资工作实效。

　　加快推进棚改旧改。注重连线成片、集中突破，推行政府购买棚改服务新模式，新开工五里牌坊、泺口、海晏门、刘智远、大庙屯、二药厂片区等58个棚户区改造项目，完成国家和省下达的45204户安置房开工任务。科学制定征地拆迁计划，加快推进东客站、济青高铁高速、轨道交通沿线、二环快速路等重点片区征地拆迁工作。严格落实区级主体责任，打好政策组合拳，建立健全服务安置居民信息平台，搭建房企与安置居民间供求桥梁，强化政策宣讲和法律服务，扎实细致做好群众工作，为城市发展、改善民生拓展空间。

　　扩大新消费新需求。支持电子商务新业态发展，加快建设国家电子商务示范城市和信息惠民示范城市，提升山大路、智汇蓝海等5个专业电商园区，推动菜鸟物流、淘宝网“特色济南馆”项目落地。制定鼓励新能源汽车发展、消费的政策措施，支持信息、绿色新消费。大力发展健康养老、教育培训、家政服务等生活性服务业，保护中华老字号。推进泉城国际旅游标志区和环城游憩带建设，力争千佛山风景名胜区成为国家级风景名胜区和5A级旅游景区，加快山东设计创意产业园、鹊山龙湖、兴隆泛旅游综合体、小清河源头湿地等文化旅游项目建设，培育百年商埠、印象济南、百花洲、火车站北广场等特色商业街区。办好第十七届中国美食节。

　　（三）补齐短板、优化提升，推动城乡区域协调发展。坚持把“三农”工作作为重中之重，同时更加重视做好城市工作，促进以人为核心的新型城镇化，加快城乡协同融合发展。

　　优化市域发展布局。加快城市更新发展，开展城市中心、次中心和卫星城规划研究, 促进城市带状格局向组团式格局转变。编制中心城区53个片区控制性详规，完成历史文化名城保护规划、历史街区保护规划报批。高标准策划黄河堤防深度开发利用，启动济南新区申报工作，加快黄河公铁大桥、长清黄河大桥建设，解决济南牌照车辆过黄河收费问题，推进北跨发展取得重要进展。

　　稳步推进新型城镇化。出台实施《济南市新型城镇化规划》。统筹推进户籍制度改革和以满足新市民需求为出发点的住房制度改革，探索实施积分落户制度，有序推进农业转移人口市民化。加大棚改旧改货币化补偿安置力度，发展住房租赁市场，改善住房供给结构，多措并举加快房地产去库存。抓好章丘国家级新型城镇化综合试点工作。实施省级示范镇提升行动，拓展镇村经济业态，稳妥推进农村社区建设，打造一批各具特色的强镇、名村。推动美丽乡村建设形态内涵双提升，注重保留农村特色、田园风光、乡土气息，完成130个示范村创建任务。通过强化产业支撑和公共服务，加快就地城镇化步伐。

　　加快城乡基础设施互联互通。推进石济客专、济青高铁、济东高速、济青高速扩容、青兰高速等项目建设，启动济泰高速、济乐高速南延、绕城大东环线建设前期工作，开工东客站客运枢纽工程，做好省会城市群经济圈城际轨道交通规划策划。实施370公里农村公路建设改造，完成972公里县乡公路安全风险路段整治。加快城市综合管廊建设，实施一批水电气热等配套设施建设工程，重点推进东区、旅游路、南康水厂建设，实施二次供水设施改造，解决270多个城中村和400多个企事业单位供水问题，巩固提升农村饮水安全工程；推进工业余热利用和腊山热源厂建设，新增集中供热面积600万平方米；加快济南高压天然气外环管网工程以及东部天然气应急调峰储配供应基地、104国道天然气应急调峰储配中心建设。加强城乡信息基础设施建设，实施200项智慧泉城建设工程，实现城乡光纤宽带全覆盖，加快“无线济南”建设。

　　打好治堵攻坚战。加快轨道交通R1线建设，开工R2、R3线一期，尽快启动轨道交通环线前期工作，完成轨道交通二期建设规划报批。加快构建高快一体快速路网，启动工业北路快速路建设，确保二环快速路、顺河高架南延2017年底全部建成通车。加快凤凰路南北段、旅游路东西段等一批主次干道建设改造，打通一批断头路。加快公交都市建设，开工龙洞、省立医院东院、高新区实训基地、唐冶公交换乘枢纽，新开通和优化调整公交线路20条以上，建设二环路无轨电车环形走廊，提高公交出行分担率。完善交通管理，启动公共自行车租赁系统建设，加强公共停车场建设管理。

　　统筹县域经济和农业农村发展。全面落实县域差别化扶持政策，以产业为纽带强化区县对接帮扶，完善市域内企业项目迁移财税利益分享机制，加速城区产业转移县域，促动园区创新转型，加快培育新机制、新空间、新项目、新动力，做大县域主导产业。大力发展现代农业，落实藏粮于地、藏粮于技战略，推进粮食高产示范区创建，实施农业科技创新与推广能力建设工程。抓好“一区六园”以及海吉星农产品物流园等现代农业综合体建设，扶持种苗花卉、中草药、茶叶、食用菌等特色农业发展。深入推进农产品质量安全五大体系建设。融合发展休闲农业与乡村旅游，重点打造4条现代农业精品示范线路和齐鲁风情8号线、五彩乡村等6条美丽乡村旅游线路。继续抓好温泉旅游以及阿胶、玫瑰等县域特色产业发展。实施新型农业经营主体培育“630”工程，重点打造6大类180个新型农业经营主体。实施“互联网+农业”行动计划，打造农产品电商平台和营销网络，培育一批特色电商镇、电商村。

　　精准务实推进脱贫攻坚。把脱贫攻坚作为第一民生工程，按照底线思维和兜底要求落实好低保、教育、医保、危旧房改造等相关政策。以产业扶贫为主攻方向，实施特困村扶贫解困示范工程，实行市级领导帮挂重点乡镇、县级领导帮挂重点村、第一书记全覆盖、一户一策一干部等措施，通过精准帮扶促进贫困地区民生改善。发挥财政投入主导作用，引导社会力量参与扶贫开发。实施金融扶贫、光伏扶贫等项目，探索利用专项扶贫资金购买社会服务。全年完成200个村5万人以上脱贫任务。

　　（四）加强生态文明建设，营造优美宜居的城乡环境。巩固国家卫生城市创建成果，采取更加科学有效的措施，强化环境治理，筑牢生态屏障，不断提高城乡宜居性和可持续性。

　　狠抓雾霾源头治理。深入推进大气污染防治“十大行动”，实施燃煤、工业、扬尘、尾气等污染协同治理，限期治理钢铁、水泥、石化等高污染行业，加快推进济钢、蓝星、庚辰等企业搬迁改造，完成东部老工业区38家企业搬迁改造和关停腾退。推进锡盟—山东特高压、榆横—潍坊特高压等“外电入鲁”项目建设，出台低谷电价等系列支持政策。加速煤改气、煤改电，实施锅炉淘汰、燃煤超低排放改造和煤炭散烧整治，全部淘汰改造建成区134台35吨/小时以下锅炉，市域内加油站全面供应国V车用汽柴油。完成既有建筑节能改造140万平方米。加强建筑垃圾和秸秆资源化利用，开展油烟污染和燃煤质量专项整治。强化区域联防联控，修订大气污染防治条例，实施环境网格化管理和24小时巡查监督行动，依法打击各类环境违法行为。

　　打造泉城特色水环境。编制名泉保护专项规划，开展白泉泉群景观恢复、9处泉域重点渗漏带保护、城区自备井全面关停、72名泉视频监测等专项行动，续建一批泉水直饮点，启动名泉博物馆规划建设，加快泉水申遗，推出一批水文化产业项目，办好第四届泉水节。推进以城市水系、园林绿地、道路交通、建筑小区为重点的43个海绵城市建设项目。开展建成区黑臭水体治理，启动腊山污水处理厂和一批分散式污水处理站建设，加快玉符河、巨野河、锦绣川、锦阳川等河道整治，推进小清河源头综合治理。加快白云湖、玫瑰湖、小清河3处国家湿地公园和14处省、市级湿地公园建设。实施东湖水库输水工程，对147座小水库小塘坝除险加固。全面完成水生态文明试点市创建任务。

　　强化生态基础建设。深化国家级生态城市和生态园林城市创建，开展城市绿荫行动，改造提升一批城市绿地和老旧公园，抓好裸露土地绿化。实施铁路沿线综合整治。加快野生动物世界二期和跑马岭生态休闲度假区建设，完成中心城区防灾避险公园建设和英雄山风景区整治。划定山体保护红线，完成26座城区山体绿化提升和山体公园建设，实施万灵山、西蒋峪等矿山地质地貌景观恢复工程。巩固提升国家森林城市创建成果，新造林5万亩、育苗2万亩，启动郊野公园建设，森林覆盖率稳定在35%以上。加大生态县和生态镇、村创建力度，建成2个国家级生态县（市）。

　　加强南部山区生态保护。完善管理体制，组建专门管理机构，实行以生态保护为主导的绩效评价考核机制，健全生态补偿机制。划定山体、河流水系、泉水重点渗漏带及泉水直接补给区四条生态控制线，统筹生态保护、产业发展、旅游、镇村建设等专项规划。做好水源涵养、生态修复、环境综治等工作，强化道路交通、污水处理和垃圾清运等基础设施建设。实施生态富民工程，高标准规划建设一批旅游、养老、休闲产业和农家乐项目，促进南部山区绿色发展、有序发展。

　　（五）实施改革开放新举措，持续增强发展动力和活力。坚持以改革解难题、以开放促发展，营造良好的体制环境。

　　加快重点改革攻坚落地。深入推进简政放权，加快政务服务大厅功能升级，动态调整市级行政审批事项目录、权力清单、责任清单，推行行政审批业务手册、服务指南“两个规范”，公开公共服务事项目录，坚决砍掉各类无谓的证明和繁琐的手续。建设事中事后监管信息化平台。开展综合行政执法体制改革，进一步减少层次、整合队伍、提高效率。推进财税改革，做好政府债务存量置换工作，深化政府事权和支出责任划分改革，搭建综合涉税信息管理应用平台，健全财金控股集团和产业发展集团市场化运作机制，加强财政预算绩效管理。推动开发区改革创新，实施《关于进一步推动省级以上开发区改革发展的指导意见》和《开发区发展三年行动计划》，做好新材料产业园和商河经济开发区改革试点工作。分类分层推进国企改革，因企制宜发展混合所有制。全面完成市政公用企业改革改制，推进公共产品供给价格改革。帮助企业降低制度性交易成本、企业税费、社会保险费、财务成本、电力价格、物流成本。完善促进民营经济发展的意见，推进中小企业信用担保体系建设，实现民营经济比重提高1个百分点。创新城市基础设施资金保障机制，通过发行城市基础设施建设企业债券、金融租赁及PPP模式，拓宽融资渠道。改革完善不动产统一登记制度。推进生产经营类事业单位转企改制取得实质进展。

　　推动全方位开放发展。实施济南城市国际化战略，积极争取国家第一批构建开放型经济新体制试点城市。落实济南市对接“一带一路”行动方案，开拓新兴市场，推动我市企业“走出去”。实施科技对外开放工程，主动融入与东盟、南亚、非洲、拉美等国家的科技伙伴计划，深化国内外科技合作。推进济南新材料产业园与德国新材料产业集群、中小企业创新园区产业合作，加快中日韩尖端产业融合园、中新智慧城等项目建设。规划建设临空经济示范区，积极开通洲际航线。发挥国际离岸服务外包优势，着力发展信息技术服务、工业工程设计等领域服务外包。积极申请加入中国（山东）自由贸易试验区，依托综保区、空港功能区，发展飞机融资租赁、航空物流等产业，强化山东一达通、秦工国贸等跨境电商综合服务平台作用，提升中小外贸企业对外贸易便利化,大力发展B2B和B2C业务，扩大网上进出口。办好第五届中国（济南）韩国商品博览会暨首届中日韩精品展，策划开展“济南制造”东盟推介会和友城行经贸活动。开工建设国际学校和国际医院。

　　（六）坚持共享发展理念，办好社会事业和民生实事。不断强化民生保障，确保各项社会政策能够托底。今年重点办好15件民生实事。

　　促进就业推动创业。重点抓好大学毕业生、就业困难人员、产业转型和化解过剩产能产生的再就业人员、城郊被征地农民等群体就业。逐步解决“蜗居”大学生就业创业和居住落户难题。完善“创业培训+创业预孵化+初创孵化+创业园区”模式，加强社会组织服务（孵化）平台建设。确保城镇新增就业10万人，农业富余劳动力转移就业5万人。

　　提升社会保障水平。做好企业退休人员基础养老金调整工作，提高城乡居民医疗保险财政补助标准。实施职工大病医疗保险和长期护理保险制度。做好低保户、五保户、零就业家庭、因病致贫家庭等特殊困难群众的生活救助工作。完善困难残疾人生活补贴和重度残疾人护理补贴制度。编制养老服务设施专项规划，支持社会力量兴办养老机构，推动医疗卫生与养老服务融合发展。推进机关事业单位养老保险制度改革。鼓励符合公租房申请条件的家庭通过市场承租住房，提高补贴标准。

　　深入推进教育均衡和教育公平。实施第二期学前教育三年行动计划。巩固“零择校”成果，完善普通高中招生政策，新建、改扩建49所中小学校，逐步解决大班额问题。完善集团化发展、委托管理、政府购买服务等多元办学机制，加大贫困家庭学生资助力度，保障农民工随迁子女平等入学。实施“技能兴济”工程，加大对中职院校和技工院校建设扶持力度。

　　提升医疗卫生服务水平。持续推进健康城市建设。深化医药卫生体制改革，全面推行城市公立医院综合改革。着力落实社会办医政策，推动医疗资源向基层、农村流动，提高基层医疗卫生服务能力。做好市中医东院区新建和市传染病医院迁建工程。实施“智慧健康”工程。稳妥落实“全面两孩”政策。

　　深入推进文化惠民。持续开展创建文明城市工作，大力培育和践行社会主义核心价值观。加强公共文化服务体系建设，组织文化惠民交流演出季，开展“齐鲁农家沁书香”活动。实施城子崖、大辛庄、齐长城遗址公园建设和“乡村记忆”工程。承办好第四届中国非物质文化遗产博览会。推进济南市博物馆新馆规划建设。实施“文化+”行动计划，推动文化创意和设计服务与旅游、商贸、教育、制造业、农业融合发展。广泛开展全民健身运动，大力发展体育产业。

　　加强和创新社会治理。健全新型社会组织体系。加强城市精细化、标准化、法治化管理，实施60个老旧小区综合整治，严格规范物业管理服务，构建优美和谐社区。扎实推进平安济南、法治济南建设，开展“法治六进”活动，搞好普法宣传教育，完善人民调解、行政调解、司法调解联动体系，构建以信息化为支撑的社会治安立体防控体系。完善社会征信系统，强化重点领域诚信建设。推行安全生产网格化管理，做好重点行业、重点区域监管和专项治理。积极创建国家食品安全城市，推进“食安济南”建设。进一步发挥12345等市民服务热线为民服务的作用。配合国防和军队建设改革，做好相关承接协调工作，积极稳妥安置军队转业人员。建设市人防指挥中心。继续做好民宗、仲裁、侨务、老龄、妇女儿童等工作。

　　各位代表，做好今年工作，任务非常艰巨，使命非常光荣。全市各级政府工作人员要自觉践行“三严三实”，坚持“实在实干实绩”工作导向，敢于责任担当，勇于干事创业，努力把全市改革发展稳定各项工作落地抓实。要按照建设法治政府的要求，提高依法办事和服务发展的能力，注重科学性和可操作性，运用法治思维、专业能力、市场办法来谋划全局、制定政策和推动工作。要发扬钉钉子精神，锲而不舍打基础谋长远，全力以赴破难题解矛盾，确保关键环节和重要工作实现突破进展。要坚持廉政勤政，摒弃为官不为，持之以恒落实中央八项规定精神，严格落实党风廉政建设责任制。进一步加强对财政资金分配使用、国有资产监管、政府采购、公共工程建设等重点领域和关键环节规范管理，严控政府债务，及时收回基金结余和沉淀资金，把更多财力用于保障和改善民生。强化审计、监察、督查等部门监督作用，自觉接受群众监督和舆论监督，自觉接受市人大及其常委会法律监督、工作监督和市政协民主监督，努力办好人大议案、建议和政协建议案、提案。

　　各位代表，承载着700多万泉城市民的梦想和期盼，我们踏上了“十三五”发展的新征程。让我们紧密团结在以习近平同志为总书记的党中央周围，在省委省政府和市委坚强领导下，解放思想，锐意进取，凝心聚力，真抓实干，认真做好“打造四个中心，建设现代泉城”各项工作，为率先实现全面建成小康社会宏伟目标而努力奋斗。
